# Late Gestation Maternal Nutrition Has a Stronger Impact on Offspring Liver Transcriptome than Full-Gestation Supplementation in Beef Cattle

**DOI:** 10.3390/vetsci12050406

**Published:** 2025-04-26

**Authors:** Guilherme Henrique Gebim Polizel, Maria Elis Perissin dos Santos, Aline Silva Mello Cesar, Wellison J. S. Diniz, German D. Ramírez-Zamudio, Paulo Fantinato-Neto, Arícia Christofaro Fernandes, Barbara Carolina Teixeira Prati, Édison Furlan, Gabriela do Vale Pombo, Miguel Henrique de Almeida Santana

**Affiliations:** 1 Department of Animal Science, Faculty of Animal Science and Food Engineering, University of São Paulo, Av. Duque de Caxias Norte, 225, Pirassununga 13635-900, SP, Brazilmhasantana@usp.br (M.H.d.A.S.); 2 Department of Food Science and Technology, Luiz de Queiroz College of Agriculture, University of São Paulo, Av. Pádua Dias 11, Piracicaba 13418-900, SP, Brazil; 3 Department of Animal Sciences, College of Agriculture, Auburn University, Auburn, AL 36849, USA; 4 ÁPIS Serviços Veterinários LTDA, R. Marques de Tamandaré, 130, Jardim Elite, Pirassununga 13636-350, SP, Brazil

**Keywords:** energy metabolism, fetal programming, gene expression, immune system, thiamine metabolism

## Abstract

Maternal nutrition’s impact on liver transcriptome in beef cattle offspring is still underexplored. We investigated the long-term effects of maternal nutrition strategies on the liver transcriptome of pre-slaughter Nelore bulls. Pregnant cows were divided into three groups, each receiving different nutritional regimens: NP (control, only mineral supplementation), PP (late gestation protein–energy supplementation), and FP (protein–energy supplementation throughout pregnancy). Liver samples were collected from male offspring aged 22.5 ± 1 months and analyzed using RNA-Seq (n = 5 per treatment). Principal component analysis (PCA) and differential gene expression analysis were carried out in an R statistical environment. Genes were considered significant when FDR < 0.05. The over-representation analysis (ORA) was performed using the clusterProfiler package from R. Metabolic pathways were considered significant when the Q-value < 0.1. The PCA showed overlapping clusters among the groups. We identified 16 differentially expressed genes (DEGs) associated with PP × NP contrast, four with FP × NP, and two with FP × PP. The ORA revealed two significant pathways (thiamine and butanoate metabolism). The identified genes and pathways were associated with vitamins, energy, oxidative metabolism, and immune function. This study emphasizes the more significant long-term effects of the PP treatment on the offspring’s liver transcriptome compared to the FP treatment.

## Simple Summary

This study explored how the diet of pregnant cows affects the biology of their calves later in life, focusing on their liver function. We compared three maternal diet strategies: one providing only basic minerals, another adding extra protein and energy during the final months of pregnancy, and a third adding protein and energy throughout pregnancy. The calves’ liver samples were analyzed as young adults to understand how their mothers’ diets influenced them. The study found that calves whose mothers received additional nutrition during late pregnancy showed the most significant changes in genes related to vitamin use, energy production, and immune response. These findings suggest that focusing on maternal nutrition during late pregnancy can have lasting effects on the health and metabolism of the offspring’s liver. This information is valuable for farmers and scientists aiming to improve animal health and efficiency in livestock production.

## 1. Introduction

Maternal nutrition is one of the primary environmental factors that influence fetal development. Epidemiological studies in humans have shown that maternal nutritional insults have consequences on the birth weight of offspring and the incidence of metabolic diseases later in adult life, establishing the foundations of what is now known as developmental programming or fetal programming [1,2]. Subsequently, studies across various mammalian species were conducted to understand fetal responses to the prenatal maternal environment [3,4,5,6,7]. These investigations deepened understanding of how maternal factors, such as diet and stress, influence fetal development and the long-term health of the offspring. In beef cattle, it has been identified that environmental factors or insults during pregnancy, especially maternal nutrition, can influence the programming of offspring traits in utero [8,9,10] and have consequences in early postnatal life [11,12]. However, the long-term effects of maternal nutrition on beef cattle offspring, particularly about changes in the genetic profile that may affect hepatic metabolism, remain less understood.

A supplementation model with vitamins and minerals, combined with different rates of weight gain during early gestation in beef heifers, can influence the hepatic metabolic profile of the offspring, mainly lipid metabolism [13]. In the same nutritional model of the dams at the beginning of gestation, Anas et al. [14] observed the effects on gene expression and the lncRNA gene network in the fetal hepatic transcriptome, affecting various pathways and biological processes related to energy and amino acid metabolism. Maternal nutrition immediately affects the offspring’s hepatic metabolism, as evident during in utero life in the mentioned studies. However, the effects of maternal nutrition at different stages of gestation on the metabolism and molecular regulation of hepatic functions in offspring over the long term during postnatal life are still poorly understood. A previous study from our laboratory demonstrated that nutritional strategies applied at different stages of gestation can impact the hepatic metabolomic profile, particularly in pathways related to oxidative and amino acid metabolism of the offspring in adult life [15].

The liver is the primary metabolic organ and performs multiple essential functions in cattle. It is especially crucial in energy metabolism, processing bilirubin, bile acids, and xenobiotics, and playing a significant role in protein synthesis and immunological function [16]. Specifically in ruminants, the liver plays a key role in nitrogen metabolism by converting ammonia nitrogen into urea for excretion or recycling, helping maintain nitrogen balance [17]. It also supports essential metabolic processes, including gluconeogenesis, fatty acid oxidation, and regulation of blood glucose, which are crucial for the health and productivity of beef cattle [18,19]. The liver is also directly related to economically important production traits, such as feed efficiency [20,21,22], which directly impacts how effectively beef cattle convert feed into body mass and overall productivity. Therefore, the liver is a vital target organ for improving cattle performance and reducing feeding costs, thereby enhancing economic returns for producers. Recently, the immediate effects of maternal nutrition on the offspring’s liver in beef cattle have been investigated following the application of nutritional approaches in the dam [23,24,25,26]. However, the molecular mechanisms and hepatic metabolic pathways in offspring affected by maternal nutrition are still poorly explored in the literature, especially their long-term impacts on the adult life of the offspring.

This study hypothesized that nutritional approaches applied at different stages of gestation have long-term effects on the liver transcriptome of bulls, impacting gene expression up to the point of slaughter (676 ± 28 days of age). The objectives were to evaluate whether the maternal nutrition at distinct stages of gestation produces lasting effects on the liver transcriptome using unsupervised methods (principal component analysis) and supervised methods (differential expression analysis) and also to understand the metabolic pathways involved.

## 2. Method

### 2.1. Experimental Design

This study is part of a broad research project aiming to unveil the long-term molecular effects of maternal nutrition on beef cattle offspring. The animals utilized in this study were all sourced from the School of Animal Science and Food Engineering (FZEA-USP) campus facilities. This study was approved by the Research Ethics Committee of the FZEA-USP, under protocol No. 1843241117, in compliance with Animal Research: Reporting of In Vivo Experiments (ARRIVE) recommendations.

Most of this section provides background information on the nutritional treatments the 126 Nelore cows received during gestation to contextualize the experimental conditions. The cows underwent artificial insemination with semen from four sires, and their pregnancies were confirmed after 30 days. The dams were grouped into three blocks of 42 animals each, according to their age (NP = 3.37 ± 1.41 years; PP = 3.59 ± 1.41 years; FP = 3.62 ± 1.49 years), body weight (BW; NP = 461.1 ± 44.7 kg; PP = 451.2 ± 60.8 kg; FP = 454.1 ± 56.7 kg), and body condition score (NP = 4.50 ± 0.58; PP = 4.60 ± 0.77; FP = 4.50 ± 0.58), to achieve a balanced experimental design. The groups were then housed in grazing paddocks of *Urochloa brizantha* cv. Marandu, with troughs provided for water and feed supplementation. The cows received the following prenatal nutrition treatments: (1) NP (control)—Not Programmed; (2) PP—Partial Programming; and (3) FP—Full Programming. The NP cows were provided solely with mineral supplementation at 0.3 g/kg of their daily BW during pregnancy. In contrast, the PP group received protein–energy supplementation at 3 g/kg of their BW daily during the third trimester, while the FP group received the same protein–energy supplementation (3 g/kg of their BW per day) from pregnancy confirmation until calving. Mineral supplementation was consistent across all groups (0.3 g/kg of BW per day) as it was included in the formulation of the protein–energy supplement (Table 1). The nutritional quality of the grazing paddocks (*Urochloa brizantha* cv. Marandu) was assessed throughout the pregnancy, revealing similar results among the groups. Specifically, the total digestible nutrients (TDNs) values were 63.07% for the NP group, 64.1% for the PP group, and 61.43% for the FP group, while the crude protein (CP) values were 7.38%, 7.82%, and 7.40% for the NP, PP, and FP groups, respectively. Further details on pasture conditions and the effects of the different maternal nutritional treatments (NP, PP, and FP) on the dams’ performance and metabolism can be found in our previous study [27].

After calving, protein–energy supplementation stopped, and all offspring (male = 65 and female = 61), regardless of maternal nutrition, followed the same health and feeding protocols until weaning (240 ± 28 days). From calving to weaning, cows received mineral supplementation (0.3 g/kg BW) and were raised in an extensive grazing system with *Urochloa brizantha* cv. Marandu pastures. This study focused exclusively on male offspring, aligning the experimental design with the management and production practices for this category. Post-weaning, animals were separated by sex and reared until 570 ± 28 days. Young bulls received distinct supplements according to the season: an energetic one in the dry season and a protein supplement in the wet season while grazing on the same pastures. The finishing phase began at 570 ± 28 days for 63 bulls (two male calves were lost during the backgrounding phase), lasting until slaughter at 676 ± 28 days, during which they followed three different diets: an adaptation diet for 15 days, a subsequent diet for 35 days, and a final diet for 56 days [15]. Following the experimental period, the animals were slaughtered at the FZEA/USP school slaughterhouse, adhering to the Brazilian Ministry of Agriculture, Livestock, and Supply standards.

### 2.2. Liver Tissue Sample Collection

All bulls were slaughtered, and liver samples were collected. For transcriptomic analysis, liver tissue samples were randomly selected from five animals per treatment group (NP [n = 5], PP [n = 5], FP [n = 5]). These samples were taken from the distal part of the left lobe, rapidly frozen in liquid nitrogen, and stored at ultra-low temperatures (−80 °C) until further analysis.

### 2.3. RNA Extraction, Processing, and Sequencing

RNA was extracted using the TRIzol reagent (Life Technologies, Carlsbad, CA, USA) following the manufacturer’s instructions. A 100 mg liver tissue sample was used for RNA isolation, and the quantity of RNA was measured with a DS-11 spectrophotometer (Denovix, Wilmington, DE, USA). RNA integrity was evaluated using the Bioanalyzer 2100 (Agilent, Santa Clara, CA, USA), yielding an average RNA integrity number (RIN) of 7.1.

For library preparation, 0.1–1 µg of RNA was utilized according to the TruSeq Stranded mRNA Reference Guide (Illumina, San Diego, CA, USA). Library quantification was performed by quantitative PCR using the KAPA Library Quantification kit (KAPA Biosystems, Foster City, CA, USA), and the average library size was determined via the Bioanalyzer 2100 (Agilent, Santa Clara, CA, USA). Sequencing was carried out on a single flow-cell lane with the TruSeq PE Cluster kit v3-cBot-HS (Illumina, San Diego, CA, USA) and a paired-end sequencing approach. The HiSeq2500 platform (Illumina, San Diego, CA, USA) was used for sequencing, and the TruSeq Stranded mRNA kit was employed according to the manufacturer’s instructions. The sequencing process was managed by NGS Soluções Genômicas (Piracicaba, SP, Brazil).

### 2.4. RNA-Seq Data Filtering and Alignment

The quality of the raw RNA-seq data was initially assessed using the FASTQC tool (version 0.11.9). Adapter sequences and low-complexity reads were removed with the SeqyClean program (version 1.9.10) [29]. Cleaned reads were then aligned to the Bos taurus ARS-UCD1.3.113 reference genome using the STAR aligner (version 020201) [30].

A count per million (CPM) table was created by removing genes that had no expression (zero counts), low expression (less than one count per million on average per sample), and genes with fewer than ten counts in at least three samples. In the end, 15,644 genes were included in the dataset following final filtering and normalization.

### 2.5. Principal Component Analysis, Differential Gene Expression, and Functional Enrichment Analysis

All the statistical analyses described below were conducted after the filtering steps detailed in Section 2.4. Principal component analysis (PCA) and differential expression analysis were performed in the R version 4.4.0 statistical environment. PCA was performed using the “prcomp” function from the base R package stats version 4.4.0. PCA was employed for two main objectives: (1) for dimensionality reduction and (2) for assessing the clustering of samples based on prenatal nutrition group. Differential expression analysis was performed using the DESeq2 version 1.44.0 [31]. Genes with an FDR < 0.05 were considered differentially expressed between the maternal nutrition groups. The differentially expressed genes (DEGs) were submitted to over-representation analysis (ORA) to uncover associated metabolic pathways impacted by maternal nutrition. DEGs from each contrast (PP × NP, FP × NP, and FP × PP) were used as input to evaluate the impact of maternal nutrition on metabolic pathways in the liver. ORA was conducted using the “enrichKEGG” function from clusterProfiler version 4.12.6 [32] to identify over-represented KEGG (Kyoto Encyclopedia of Genes and Genomes) metabolic pathways. Pathways were considered significant when the Q-value was < 0.1.

## 3. Results

### 3.1. Principal Component Analysis (PCA)

**Figure 1:**
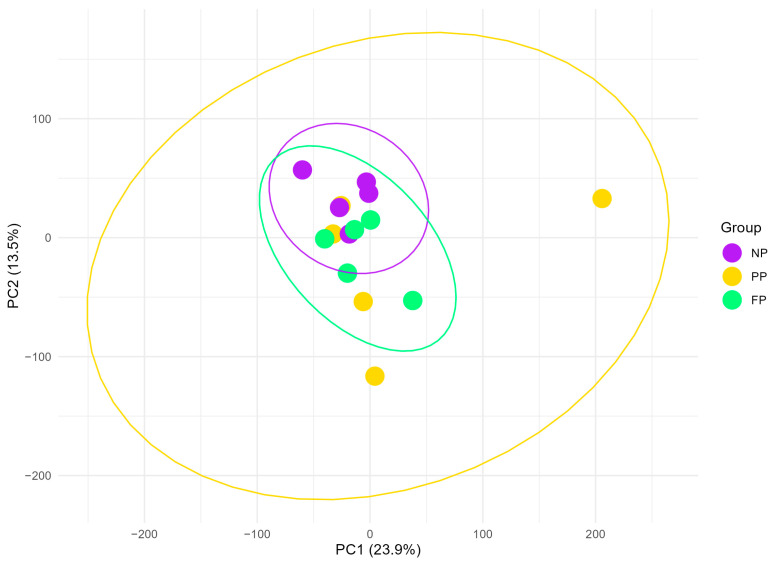
Principal component analysis (PCA) plot of liver transcriptome profiles in bulls during finishing phase, grouped by the maternal nutritional groups (NP, PP, and FP). Ellipses (yellow, green, and purple) represent 95% confidence intervals around the mean PCA scores for each group, illustrating the variability and clustering of transcriptome profiles within each maternal nutritional group.

The data dimensionality reduction performed by PCA (Figure 1) revealed that the PP group exhibits the most dispersed clustering, encompassing the NP and FP groups entirely, which may reflect greater transcriptomic variability within this group. In contrast, the NP and FP groups show less variability in their clustering, with partial overlap between them, indicating a closer transcriptomic relationship than their respective relationships with the PP group (PP × NP and FP × PP). The FP and PP groups appear highly similar, as three of the five PP samples fall within the FP cluster. The first two principal components accounted for 37.4% of the total variance (PC1 = 23.9%; PC2 = 13.5%).

### 3.2. Differentially Expressed Genes (DEGs)

**Figure 2:**
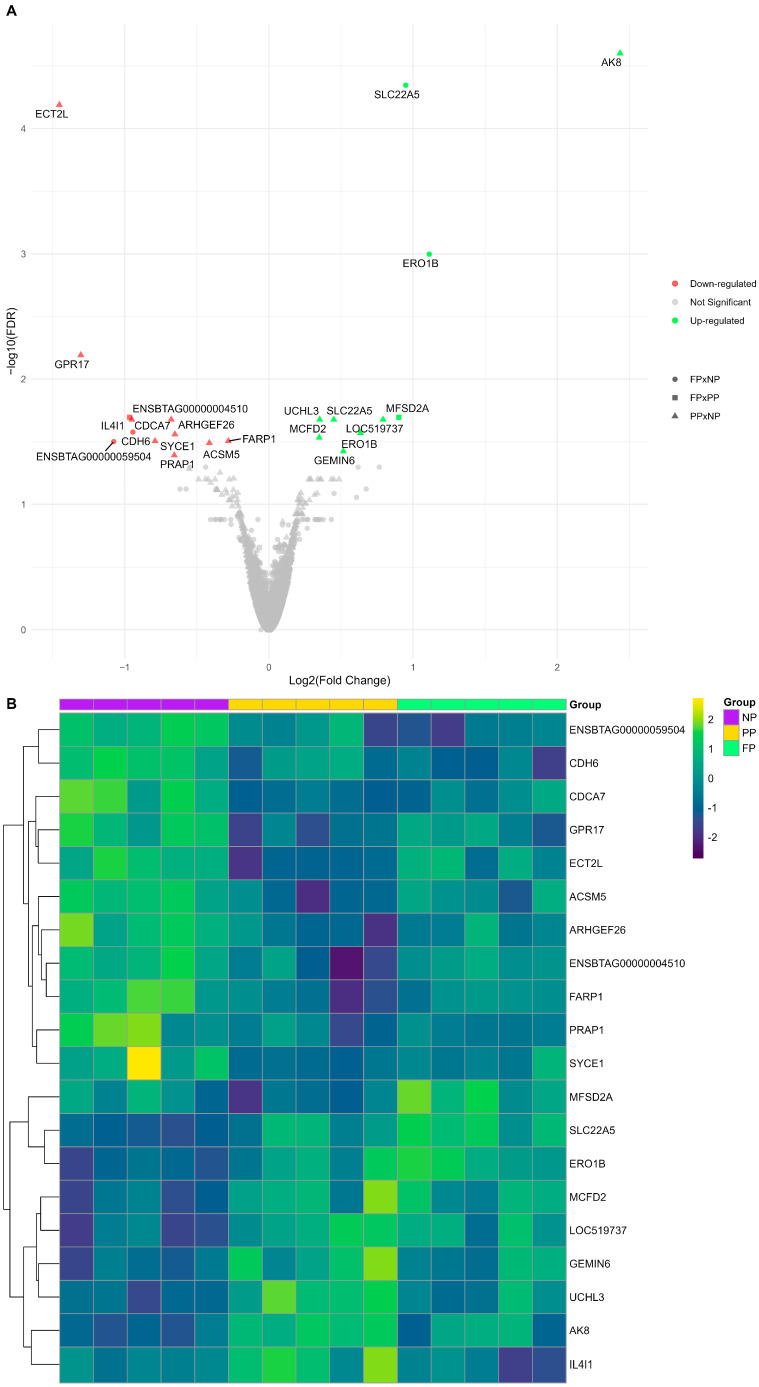
(**A**) Volcano plot illustrating each maternal nutrition group contrast, where downregulated genes are shown in red, upregulated genes in green, and non-significant genes are shown in gray. The PP × NP contrast is represented by triangles, the FP × NP contrast by circles, and the FP × PP contrast by squares. (**B**) Heatmap of log2(CPM) transformed gene expression levels for the differentially expressed genes (DEGs) among the prenatal nutritional groups (NP, PP, and FP).

We identified 22 DEGs (FDR < 0.05) among the three different maternal nutrition group contrasts (PP × NP, FP × NP, and FP × PP; Figure 2). The PP × NP contrast demonstrated nine downregulated genes (*ECT2L*, *PRAP1*, *ACSM5*, *FARP1*, *SYCE1*, *ARHGEF26*, *CDCA7*, *ENSBTAG00000004510*, and *GPR17*) and seven upregulated genes (*AK8*, *GEMIN6*, *MCFD2*, *ERO1B*, *SLC22A5*, *UCHL3*, and *LOC519737*). The FP × NP contrast showed two downregulated genes (*CDH6* and *ENSBTAG00000059504*) and two upregulated genes (*SLC22A5* and *ERO1B*), while the FP × PP contrast revealed only one upregulated gene (*MFSD2A*) and one downregulated gene (*IL4I1*). The genes *SLC22A5* and *ERO1B* were common to both PP and FP, showing upregulation in the treatments with protein–energy supplementation compared to the control (NP).

The impacts of maternal nutrition on the liver transcriptome are shown by the visual representation of the log_2_(CPM) gene expression patterns of the DEGs (Figure 2B). The results reporting all genes and their respective statistical values are available in Supplementary Table S1 (PP × NP), Table S2 (FP × NP), and Table S3 (FP × PP).

### 3.3. Gene Functional Enrichment Analysis

**Figure 3:**
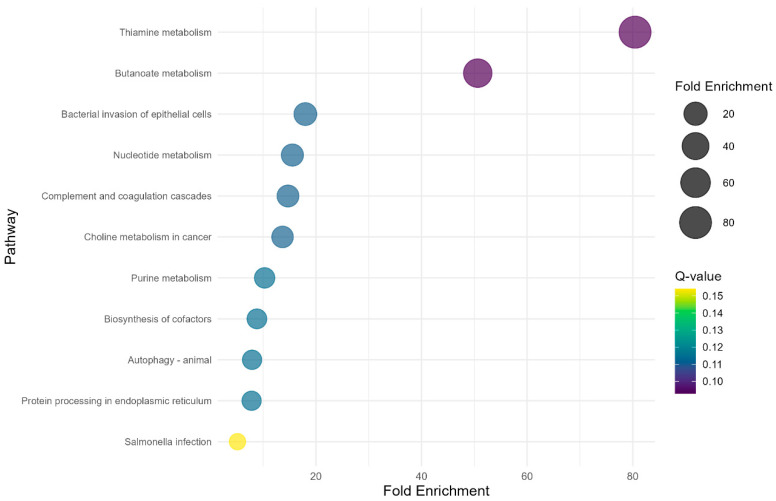
Bubble plot showing KEGG over-representation analysis of the DEGs from the PP × NP contrast.

The study identified two significant (Q-value < 0.1) KEGG metabolic pathways (Figure 3) in the PP × NP contrast. Thiamine metabolism exhibited the highest fold enrichment (80.44) with a Q-value of 0.09, while butanoate metabolism showed a fold enrichment of 50.65, also with a Q-value of 0.09. However, regarding the other contrasts (FP × PP and FP × NP) no significant metabolic pathways were found (Q-value > 0.1).

## 4. Discussion

Our findings revealed new insights into the long-term effects of maternal nutritional supplementation strategies (NP, PP, and FP) on the liver transcriptome of Nelore bulls, emphasizing both the gene expression patterns and metabolic pathways influenced by maternal diet. Throughout this discussion, we focused on the most significant DEGs in each contrast (PP × NP = *AK8* and *ECT2L*; FP × NP = *SLC22A5* and *ERO1B*; FP × PP = *MFSD2A* and *IL4I1*) and the KEGG pathways associated with the maternal nutritional treatments.

Overall, DEGs were predominantly associated with the PP × NP contrast (16 of 22), indicating that protein–energy supplementation in late gestation had a stronger impact on the liver transcriptome than supplementation throughout gestation (FP × NP = four DEGs). This greater influence of the PP treatment may be related to the timing of supplementation initiation and/or the impact of providing additional protein and energy during rapid fetal growth in late gestation [33]. Consequently, pathways related to vitamin (thiamine metabolism) and energy (butanoate metabolism) metabolism were identified in this contrast. The FP × PP contrast showed only subtle differences in the two genes, which aligns with the fact that both treatments received similar nutrition during the third trimester. Therefore, the protein–energy supplementation provided during the first six months of gestation, which differentiates FP from PP, does not appear to affect the liver transcriptome significantly.

One of the notable genes identified in this study belongs to the adenylate kinase (*AK*) family, a group of enzymes associated with the cellular nucleotide synthesis machinery [34] and catalyzer of the reaction ATP + AMP ⇌ 2ADP [35]. The *AK* gene is generally required for all aspects of an organism’s normal functioning, including growth, differentiation, motility, and energy metabolism [35]. The *AK8* gene, a member of the *AK* family, is expressed in some organs, such as the liver, pancreas, and testis, playing a negative regulatory role in epithelial cell migration [36]. Notably, a recent study in sheep showed that paternal methionine supplementation hypermethylated the *AK8* gene in sperm and exhibited a strong negative correlation (r = −0.757) with the expression levels of the *AK8* gene in embryos [37]. This implies that the paternal diet’s influence on methyl marks may be transmitted to the embryo and affect transcription efficiency. However, no studies have reported maternal nutrition’s effects on offspring *AK8* gene levels. Notably, *AK8* was the most significant gene in this study (FDR = 2.5 × 10^−5^) and exhibited the highest log fold change (2.43). Therefore, this study is the first to identify an association between maternal protein–energy supplementation during late gestation and the expression of this gene in the offspring’s liver.

The *ECT2L* gene (epithelial cell transforming 2 like) regulates Rho protein signal transduction and has been linked to both cancer progression and cholesterol metabolism [38]. Additionally, recurrent somatic mutations in *ECT2L* have been identified in early T-cell precursor acute lymphoblastic leukemia [39]. In ruminant production, this gene was found to be more highly expressed in the abomasal lymph nodes of lambs susceptible to helminth infections than those with resistance [40]. Although limited information about this gene is available in the literature, its known functions and mechanisms are associated with immune regulation. The greater expression of *ECT2L* in NP bulls suggests a potential negative impact on their immune function. In contrast, *SLC22A5* (solute carrier family 22 member 5) was overexpressed in the groups that received protein–energy supplementation (PP and FP) compared to the group that did not (NP). This gene is part of the solute carrier *(SLC)* transporter family and plays a critical role in facilitating the transport of various substances across biological membranes. It is essential for maintaining the balance of key molecules, such as inorganic ions, amino acids, lipids, sugars, neurotransmitters, and drugs, thereby supporting overall cellular function and homeostasis within the organism [41]. Specifically, *SLC22A5* gene encodes an important transporter of L-carnitine (*OCTN2*), which plays a critical role in the transport of long-chain fatty acids for β-oxidation [42] and the movement of peroxisomal β-oxidation metabolites into mitochondria for complete oxidation via the tricarboxylic acid (TCA) cycle [43,44]. This strongly supports the oxidative impact of prenatal nutrition on the liver. The upregulation of *SLC22A5* in the groups that received protein–energy supplementation (PP and FP) could be linked to increased energy production in the liver. In our previous study [45], we identified several genes from the *SLC* family, including *SLC12A8* and *SLC6A14*, as hub components in significant modules of gene co-expression analysis transcriptomics–metabolomics integrative analysis within the NP and FP groups. These findings highlight the importance of further research to explore the impacts of prenatal nutrition on this gene family and its potential implications for the oxidative metabolism and immune system.

The endoplasmic reticulum oxidoreductin 1-like beta (*ERO1B)* is another gene overexpressed in the treatment that received protein–energy supplementation (PP and FP) in comparison with the treatment that did not receive it (NP). This finding highlights the similarities in the effects of maternal protein–energy supplementation on gene expression, although the PP treatment generally exhibited a much greater influence on the hepatic transcriptome. *ERO1B* is critical in several physiological processes, including insulin biogenesis, glucose homeostasis, and maintaining redox homeostasis within the endoplasmic reticulum. By promoting oxidative protein folding and regulating redox balance, *ERO1B* directly influences oxidative metabolism, ensuring the proper handling of reactive oxygen species and the maintenance of cellular function [46,47,48]. The overexpression of this gene suggests that maternal protein–energy supplementation may enhance these processes, potentially leading to improved metabolic function in the offspring. In addition, in our previous study [15], the different maternal nutrition groups also impacted oxidative metabolism, as evidenced by metabolomics analysis of the liver in the progeny, corroborating our present findings.

Regarding the two differentially expressed genes (DEGs) associated with the FP × PP contrast, the major facilitator superfamily domain-containing protein 2 alpha (*MFSD2A*) was upregulated in the FP group. This finding highlights the role of *MFSD2A* in metabolic adaptations to fasting in the liver, which are controlled mainly by the nuclear hormone receptor peroxisome proliferator-activated receptor alpha (*PPARα*) [49]. The *PPARα* upregulates genes involved in the biochemical pathways for β-oxidation of fatty acids, ketogenesis, and gluconeogenesis [50,51]. In contrast, interleukin 4 induced 1 gene (*IL4I1*) was downregulated in the FP treatment, being strongly associated with immune regulation. Previous studies in humans [52,53] have identified a correlation between *IL4I1* and immunosuppression, mainly by suppressing T cell proliferation and differentiation. The results of this contrast (FP × PP) further corroborate the overall impact of maternal nutrition on genes associated with immune function and energy metabolism in the liver.

Finally, the KEGG pathway ORA revealed thiamine and butanoate metabolism as the significant metabolic pathways impacted by maternal supplementation during late gestation (PP × NP) in the offspring’s liver transcriptome. Thiamine, also known as vitamin B1, is a cofactor for some enzymes related to energy metabolism, playing roles in the biosynthesis of neurotransmitters, antioxidant activity, synthesis of pentoses used as nucleic acid precursors, and immune system activation [54,55]. Butanoate metabolism plays a crucial role in organisms’ energy production. It begins with butyric acid production, followed by decarboxylation and a series of synthetic enzyme reactions that ultimately generate adenosine triphosphate (ATP) [56]. The observed impact of maternal nutrition on these pathways suggests alterations in key mechanisms associated with energy efficiency. These findings align with the previous results discussed in this study, emphasizing the potential influence of maternal nutrition on crucial molecular processes in the liver.

## 5. Conclusions

The influence of maternal nutrition during late gestation (PP) on the liver transcriptome was greater than that of supplementation throughout gestation (FP) when compared to the control (NP), leading to the modulation of key metabolic pathways. While differences between the protein–energy supplementation treatments (FP × PP) and FP vs. NP were subtle, our findings provide new insights into how maternal nutrition affects offspring liver function. These results suggest that managing nutrition during late gestation could optimize beef cattle performance, highlighting the need for further studies to better understand the long-term effects of prenatal nutrition on cattle health and productivity.

## Figures and Tables

**Table 1:** Composition and nutritional content of the maternal supplement.

Ingredients	Mineral Supplement	Protein–Energy Supplement
Corn (%)	35.00	60.00
Soybean meal (%)	-	30.00
Dicalcium phosphate (%)	10.00	-
Urea 45% (%)	-	2.50
Salt (%)	30.00	5.00
Minerthal 160 MD (%) *	25.00	2.50
Total digestible nutrients (%)	26.76	67.55
Crude protein (%)	2.79	24.78
Non-protein nitrogen (%)	-	7.03
Acid detergent fiber (%)	1.25	4.76
Neutral detergent fiber (%)	4.29	11.24
Fat (%)	1.26	2.61
Calcium (g/kg)	74.11	6.20
Phosphorus (g/kg)	59.38	7.24

* Mineral premix composition (Minerthal company): calcium = 8.6 g/kg; cobalt = 6.4 mg/kg; copper = 108 mg/kg; sulfur = 2.4 g/kg; fluorine = 64 mg/kg; phosphorus = 6.4 g/kg; iodine = 5.4 mg/kg; manganese = 108 mg/kg; selenium = 3.2 mg/kg; zinc = 324 mg/kg; sodium monensin = 160 mg/kg [28].

## Data Availability

The transcriptome dataset analyzed during the current study is available in the European Nucleotide Archive (ENA) repository (EMBL-EBI), under accession PRJEB75582 (http://www.ebi.ac.uk/ena/browser/view/PRJEB75582, accessed on 20 December 2024).

## Supplementary Materials

The following supporting information can be downloaded at https://www.mdpi.com/article/10.3390/vetsci12050406/s1. Supplemental Table S1: Differential expression analysis from PP × NP contrast; Supplemental Table S2: Differential expression analysis from FP × NP contrast; Supplemental Table S3: Differential expression analysis from FP × PP contrast.

## Abbreviations

*AK*	Adenylate kinase
ARRIVE	Animal Research: Reporting of In Vivo Experiments
ATP	Adenosine triphosphate
BW	Body weight
CP	Crude protein
CPM	Counts per million
DEGs	Differentially expressed genes
*ECT2L*	Epithelial cell transforming 2 like
*ERO1B*	Endoplasmic reticulum oxidoreductin 1-like beta
FP	Full programming
*IL4I1*	Interleukin 4 induced 1
KEGG	Kyoto Encyclopedia of Genes and Genomes
NP	Not programmed
ORA	Over-representation analysis
PCA	Principal component analysis
PP	Partial programming
*PPARα*	Peroxisome proliferator-activated receptor alpha
RIN	RNA integrity number
*SLC22A5*	Solute carrier family 22 member 5
TDN	Total digestible nutrients
